# Effectiveness of home-based exercise combined with nutritional care intervention in elderly patients with gastric cancer and sarcopenia: a retrospective study

**DOI:** 10.3389/fmed.2025.1544689

**Published:** 2025-05-22

**Authors:** Chun-Jing Yang, Li Chen, Qing Zhou, Hui Hou, Feng-Yi Xie

**Affiliations:** ^1^Gastric Cancer Center, The First Affiliated Hospital with Nanjing Medical University, Nanjing, Jiangsu, China; ^2^Chinese Hospital Reform and Development Institute, Nanjing University, Nanjing, Jiangsu, China; ^3^Jiangsu Provincial People’s Hospital, Nanjing, Jiangsu, China

**Keywords:** gastric cancer, sarcopenia, home-based exercise, nutritional care, quality of life, nutritional biomarkers

## Abstract

**Background:**

Sarcopenia and malnutrition are prevalent among elderly patients with gastric cancer, significantly impairing recovery and quality of life (QoL). Effective interventions targeting both nutritional and physical deficiencies are critical. This study evaluated the effectiveness of a home-based exercise combined with nutritional care intervention in improving nutritional status, patient-generated subjective global assessment (PG-SGA) scores, and QoL among elderly patients with gastric cancer and sarcopenia.

**Methods:**

A retrospective evaluation was conducted on 126 elderly patients (aged ≥ 65 years) with gastric cancer and sarcopenia between January 2021 and December 2023. Patients were divided into two groups: the observation group (*n* = 61) received a home-based exercise combined with nutritional care intervention, while the control group (*n* = 65) received standard oncological nursing care. Nutritional status was assessed using biomarkers (albumin, prealbumin, transferrin) and PG-SGA scores, while QoL was measured using the Generic Quality of Life Inventory-74 (GQOLI-74). Data was analyzed using independent *t*-tests and Chi-square tests, with *p* < 0.05 considered statistically significant.

**Results:**

Baseline characteristics were comparable between the groups (*P* > 0.05). Following the intervention, the observation group exhibited significantly greater improvements in nutritional biomarkers compared to the control group: albumin (41.80 ± 5.45 g/L vs. 32.25 ± 5.37 g/L, *t* = 9.905, *P* < 0.001), prealbumin (288.59 ± 25.95 mg/L vs. 219.20 ± 23.05 mg/L, *t* = 15.89, *P* < 0.001), and transferrin (2.28 ± 0.28 g/L vs. 1.77 ± 0.23 g/L, *t* = 11.20, *P* < 0.001). The observation group’s PG-SGA scores decreased significantly (1.28 ± 0.28 vs. 4.33 ± 0.56 in the control group, *t* = 38.28, *P* < 0.001). QoL scores in physical, psychological, social, and material life domains also showed substantial improvements in the observation group compared to the control group (*P* < 0.001 for all).

**Conclusion:**

The home-based exercise combined with nutritional care intervention significantly enhanced nutritional status, reduced PG-SGA scores, and improved QoL in elderly patients with gastric cancer and sarcopenia. This integrated approach demonstrates its value as an effective strategy for comprehensive cancer care, addressing both physical and nutritional deficits to optimize recovery outcomes.

## 1 Introduction

Gastric cancer (GC) remains one of the most common and lethal malignancies worldwide, particularly in elderly populations. According to global cancer statistics, GC ranks as the fifth most common cancer and the third leading cause of cancer-related mortality. Despite advancements in diagnostic and therapeutic techniques, the prognosis for elderly patients with GC remains poor due to late-stage diagnoses and the frequent presence of comorbidities (1, 2). Among these, sarcopenia characterized by a progressive loss of skeletal muscle mass and strength is increasingly recognized as a significant complication and prognostic factor in elderly GC patients (3, 4). Studies indicate that sarcopenia is associated with reduced treatment tolerance, increased postoperative complications, and decreased overall survival. Consequently, addressing sarcopenia in the clinical management of GC has become an area of growing interest (5–7).

Sarcopenia is a geriatric syndrome characterized by an age-related decline in skeletal muscle mass and function, primarily resulting from chronic illnesses and compounded by factors such as inflammation, nutritional deficiencies, and reduced physical activity. In the context of gastric cancer, the condition is further exacerbated by cancer-related cachexia and treatment-induced side effects, such as malabsorption and anorexia. These factors synergistically contribute to a decline in functional status and quality of life, complicating the therapeutic journey of GC patients. The prevalence of sarcopenia in elderly GC patients is alarmingly high, and its adverse impact on outcomes underscores the urgent need for effective interventions tailored to this vulnerable population (8, 9). In recent years, home-based exercise programs have garnered attention as a feasible and effective strategy for managing sarcopenia in various clinical settings. Resistance training, in particular, has been shown to enhance muscle strength, physical function, and overall health. Furthermore, nutritional care interventions, including adequate protein intake and supplementation with essential nutrients, are critical in counteracting muscle loss and promoting muscle protein synthesis. Evidence suggests that the combination of physical exercise and nutritional support yields synergistic benefits, addressing both the physical and metabolic aspects of sarcopenia (10, 11). However, while promising results have been reported in other conditions, the application of these interventions in elderly GC patients remains underexplored.

This study aims to evaluate the effectiveness of a home-based exercise program combined with nutritional care interventions in improving outcomes for elderly GC patients with sarcopenia. Specifically, it seeks to assess improvements in muscle strength, physical function, nutritional status, and overall quality of life, providing robust evidence to guide clinical practice.

## 2 Materials and methods

### 2.1 Study design

This retrospective evaluation was conducted at our hospital to investigate the effectiveness of home-based exercise combined with nutritional care intervention in elderly patients with gastric cancer and sarcopenia. The study spanned from January 2021 to December 2023 and adhered to the STROBE (Strengthening the Reporting of Observational Studies in Epidemiology) guidelines (12). Eligible participants included patients aged ≥65 years with a histopathologically confirmed diagnosis of gastric cancer and sarcopenia, as defined by the Asian Working Group for Sarcopenia (AWGS) or the European Working Group on Sarcopenia in Older People (EWGSOP) criteria. Inclusion required reduced muscle mass, strength, and/or physical performance, sufficient functional independence to participate in a home-based exercise program, malnutrition or risk of malnutrition assessed by the Mini Nutritional Assessment (MNA), and signed informed consent. Patients were excluded if they had severe or uncontrolled comorbidities, metastatic disease, a history of major surgery unrelated to gastric cancer within 1 month prior to enrollment, recent chemotherapy or radiotherapy, contraindications to exercise, cognitive impairment, prior participation in similar interventions, or if they had incomplete clinical records or were lost to follow-up. A total of 126 patients were included, with 65 receiving standard care (control group) and 61 undergoing the home-based exercise combined with nutritional care intervention (observation group). This study evaluated clinical outcomes in both cohorts. Informed consent was obtained from all subjects and/or their legal guardian(s). The study’s protocols were rigorously reviewed and approved by the Ethics Committee of The First Affiliated Hospital of Nanjing Medical University (Approval No. 2024-SR-132; approved on July 25, 2024) and conducted in strict adherence to relevant guidelines and the Declaration of Helsinki’s ethical principles for human research. Methods were designed and implemented to ensure confidentiality, with all personal identifiers removed before analysis to safeguard participant privacy.

### 2.2 Conventional care for the control group

Patients in the control group received routine oncological nursing care, which included the following components:

Dietary Education: Upon admission, the designated nurse provided health education on dietary habits, including guidance on preoperative dietary patterns and nutritional structure.

Nutritional Support: Nutritional supplementation was tailored to the patients’ daily caloric requirements, calculated at 25–30 kcal/kg/day, helping them transition gradually to an appropriate diet.

Functional Exercise Guidance: Preoperative and postoperative functional exercises were conducted 2–4 times daily to promote recovery. Postoperatively, patients were provided with an abdominal binder within 24 h to protect the surgical incision and encouraged to engage in mobility activities. Initial steps included sitting at the bedside for 2–3 min, progressing to standing and performing 5 min of bedside activities. Daily activity levels were gradually increased based on the patient’s recovery status to enhance postoperative rehabilitation.

### 2.3 Home-based exercise combined with nutritional care intervention for the observation group

Patients in the observation group received a structured intervention integrating advanced nutritional care and resistance-based exercise tailored to their individual needs. Nutritional support for the observation group was implemented using the five-step nutritional therapy model, which stratified patients into five levels based on their nutritional needs and clinical condition. (1) Total Parenteral Nutrition (TPN): For patients with the most severe nutritional deficits, TPN was provided to ensure immediate and adequate nutrient delivery. (2) Combined Partial Enteral and Parenteral Nutrition: For patients not meeting full nutritional requirements via the enteral route alone, a combination of enteral and parenteral nutrition was administered to achieve a balanced nutrient supply. (3) Full Enteral Nutrition: Patients who were able to tolerate enteral feeding received full enteral nutrition to maintain gastrointestinal function and provide sufficient calories and nutrients. (4) Oral Nutritional Supplementation: For patients with moderate nutritional risks, oral nutritional supplements were provided to augment daily nutritional intake. (5) Standard Dietary Plan with Nutritional Education: For patients with stable nutritional status, a standard dietary plan was maintained. This plan was supplemented with comprehensive nutritional education to ensure that patients maintained a balanced diet during the recovery period.

Resistance-based exercise programs were designed to suit the patients’ physical condition, focusing on four key movements: (1) Straight Arm Chest Expansion: Patients held a resistance band with both hands, keeping elbows bent. Arms were raised forward and stretched outward to achieve *a* > 180° angle before returning to the original position. (2) Bicep Curl: The resistance band was looped under the patient’s legs, with both ends gripped firmly. Elbows were flexed to tighten the biceps, ensuring relaxed shoulders and tight upper arms during each movement. (3) Leg Press: One leg bent at the hip and knee pressed against the resistance band, while the other rested flat on the bed. The leg pressing the resistance band was extended and returned to the initial position, alternating between legs. (4) Knee Raise: The resistance band was secured to the bed frame, with one foot hooked under it. The hooked leg was raised by flexing the hip and knee, alternating between legs.

During postoperative home care, patients performed all four exercises in a seated position. Each exercise consisted of 4 sets lasting 40–60 s per set, with a 2-min rest between sets. From postoperative day 1 to day 3, patients practiced the first three exercises in a semi-recumbent position, with a 1-min rest interval between sets. This exercise regimen was continued for 3 months, with progressive adjustments to intensity and duration based on the patients’ recovery status, to maximize muscle strength, physical function, and overall rehabilitation outcomes.

### 2.4 Nutritional and quality of life assessments

Nutritional Status Assessment: The nutritional status of patients was evaluated preoperatively and at 3 months postoperatively using both laboratory biomarkers and a validated subjective assessment tool. (1) Laboratory Biomarkers: Blood samples were collected to measure albumin, prealbumin, and transferrin levels as key indicators of nutritional status. These biomarkers offered objective insights into the patients’ nutritional changes over time, reflecting their protein metabolism and overall nutritional recovery. (2) patient-generated subjective global assessment (PG-SGA): Nutritional status was further assessed using the PG-SGA, a validated tool combining self-reported and clinician-evaluated parameters. The patient-reported (PG) section included assessments of recent weight changes, dietary intake, diet-related symptoms, and activity levels. The clinician-evaluated (SGA) section encompassed disease-related factors, metabolic stress levels, and physical examination findings. The total PG-SGA score, ranging from 0 to 35, was calculated by summing the scores of both sections, with higher scores indicating poorer nutritional status.

Quality of Life Assessment: Quality of life (QoL) was evaluated 3 months postoperatively using the Generic Quality of Life Inventory-74 (GQOLI-74). This comprehensive questionnaire assessed four domains: physical function, psychological function, social function, and material well-being. The inventory consisted of 20 factors and 74 items, with each item scored on a scale of 1 to 5. Final scores in each domain were standardized to a percentage scale, with higher scores reflecting better QoL. The GQOLI-74 demonstrated high internal consistency, with a Cronbach’s α coefficient of 0.860, ensuring reliable measurements.

### 2.5 Statistical analysis

Statistical analyses were performed using SPSS software (Version 27.0). For continuous variables exhibiting a normal distribution, independent sample *t*-tests were applied to evaluate between-group differences at each time point, with the data expressed as mean ± standard deviation. In addition to these between-group comparisons, the present study accounted for within-group variations over time (pre- vs. post-intervention) by employing repeated measures analysis of variance (ANOVA). For categorical variables, frequencies and percentages were determined, and group associations were examined using the Chi-square (χ^2^) test; when the conditions for the Chi-square test were not met, Fisher’s exact test was adopted as an alternative. All statistical tests were two-sided, with a significance level set at *p* < 0.05.

## 3 Results

### 3.1 Baseline characteristics of patients in the control and observation groups

The baseline characteristics of the patients in the control (*n* = 65) and observation (*n* = 61) groups were well-balanced, with no statistically significant differences observed across all variables (*P* > 0.05). In terms of sex distribution, the proportion of males and females was similar between the two groups. The mean age was 57.02 ± 5.35 years in the control group and 56.71 ± 5.48 years in the observation group, with no significant difference (*t* = 0.321, *P* = 0.749). The Body Mass Index (BMI) and Karnofsky Performance Status (KPS) scores were comparable between the groups. The mean BMI was 20.48 ± 1.40 kg/m^2^ in the control group and 20.53 ± 1.46 kg/m^2^ in the observation group (*t* = 0.196, *P* = 0.845). Similarly, the mean KPS score was 71.12 ± 5.51 in the control group and 70.89 ± 5.47 in the observation group (*t* = 0.235, *P* = 0.815), indicating a similar baseline functional status. Regarding tumor-related characteristics, the distribution of tumor stages and locations was also comparable between the groups. The proportions of patients in Stages I to IV did not differ significantly (χ^2^ = 1.642, *P* = 0.543), with the majority of patients in early tumor stages (Stages I and II). Similarly, tumor locations were distributed across the antrum, body, and other regions without significant intergroup differences (χ^2^ = 1.732, *P* = 0.516) (Table 1). Overall, the baseline characteristics demonstrated homogeneity between the control and observation groups, ensuring the comparability of the study population and minimizing potential confounding variables.

**TABLE 1:** Baseline characteristics of patients in the control and observation groups (n, $\bar{x}$ ± s).

Variable	Control group (*n* = 65)	Observation group (*n* = 61)	χ^2^ or *t*-value	*P*-value
Sex (n)	–	–	0.001	0.968
Male	36	34	–	–
Female	29	27	–	–
Mean age (years)	57.02 ± 5.35	56.71 ± 5.48	0.321	0.749
BMI (kg/m^2^)	20.48 ± 1.40	20.53 ± 1.46	0.196	0.845
KPS score (points)	71.12 ± 5.51	70.89 ± 5.47	0.235	0.815
Tumor stage (n)	–	–	1.642	0.543
Stage I	26	25	–	–
Stage II	23	21	–	–
Stage III	10	9	–	–
Stage IV	6	6	–	–
Tumor location (n)	–	–	1.732	0.516
Antrum	29	31	–	–
Body	18	17	–	–
Other	18	13	–	–

BMI, Body Mass Index; KPS, Karnofsky Performance Status.

### 3.2 Comparison of nutritional indicators before and after home-based exercise combined with nutritional care intervention

The home-based exercise combined with nutritional care intervention significantly improved key nutritional indicators in the observation group compared to the control group, including albumin, prealbumin, and transferrin levels. At baseline, there were no significant differences between the groups, confirming comparable nutritional statuses before the intervention (*P* > 0.05). Mean albumin levels were 25.35 ± 4.87 g/L in the observation group and 25.68 ± 4.85 g/L in the control group (*t* = 0.381, *P* = 0.704). Similarly, prealbumin and transferrin levels were balanced between the groups, establishing a consistent baseline for evaluating the intervention’s impact. After the intervention, the observation group demonstrated significantly greater improvements in all nutritional parameters than the control group (*P* < 0.001 for all comparisons). Albumin levels increased markedly to 41.80 ± 5.45 g/L in the observation group, compared to 32.25 ± 5.37 g/L in the control group (*t* = 9.905, *P* < 0.001). Prealbumin levels rose sharply to 288.59 ± 25.95 mg/L in the observation group, significantly exceeding the 219.20 ± 23.05 mg/L observed in the control group (*t* = 15.89, *P* < 0.001). Transferrin levels also improved notably, reaching 2.28 ± 0.28 g/L in the observation group versus 1.77 ± 0.23 g/L in the control group (*t* = 11.20, *P* < 0.001) (Table 2). These findings highlight the effectiveness of the combined intervention in enhancing nutritional recovery. The integration of resistance-based exercise with tailored nutritional care resulted in substantial improvements in protein metabolism and overall nutritional status, demonstrating its critical role in promoting recovery and improving clinical.

**TABLE 2:** Comparison of nutritional indicators before and after intervention in observation and control groups (mean ± SD).

Indicator	Observation group (*n* = 61)	Control group (*n* = 65)	*t*-value	*p*-value
Albumin (g/L)	–	–	–	–
Before intervention	25.35 ± 4.87	25.68 ± 4.85	0.381	0.704
After intervention	41.80 ± 5.45*	32.25 ± 5.37*	9.905	<0.001
Prealbumin (mg/L)	–	–	–	–
Before intervention	145.31 ± 21.34	138.92 ± 21.30	1.681	0.095
After intervention	288.59 ± 25.95*	219.20 ± 23.05*	15.89	<0.001
Transferrin (g/L)	–	–	–	–
Before intervention	1.36 ± 0.16	1.32 ± 0.15	1.448	0.150
After intervention	2.28 ± 0.28*	1.77 ± 0.23*	11.20	<0.001

*Indicates a statistically significant difference compared to before intervention, with *p* < 0.05.

### 3.3 Comparison of PG-SGA scores before and after home-based exercise combined with nutritional care intervention

The home-based exercise combined with nutritional care intervention significantly improved PG-SGA scores in the observation group, indicating its superior effectiveness in enhancing nutritional status and overall recovery compared to conventional care. At baseline, there were no significant differences in PG-SGA scores between the observation and control groups, confirming the comparability of nutritional status prior to the intervention (7.51 ± 1.38 vs. 7.59 ± 1.31; *t* = 0.334, *P* = 0.739). This balance provided a reliable foundation for evaluating the intervention’s effects. After 3 months of intervention, the observation group exhibited a dramatic improvement in PG-SGA scores, decreasing to 1.28 ± 0.28, compared to a more modest reduction to 4.33 ± 0.56 in the control group (*t* = 38.28, *P* < 0.001) (Table 3). These results highlight the substantial benefit of integrating resistance-based exercises with personalized nutritional care in addressing nutritional deficiencies and promoting recovery. The intervention’s effectiveness is evident in its ability to achieve greater reductions in PG-SGA scores, reflecting significant improvements in nutritional status and overall well-being. This integrated approach demonstrates its potential as a comprehensive strategy for optimizing postoperative rehabilitation and enhancing patient outcomes.

**TABLE 3:** Comparison of PG-SGA scores before and 3 months after intervention in observation and control groups.

Group	Observation group (*n* = 61)	Control group (*n* = 65)	*t*-value	*p*-value
Before intervention	7.51 ± 1.38	7.59 ± 1.31	0.334	0.739
After intervention	1.28 ± 0.28*	4.33 ± 0.56*	38.28	<0.001

PG-SGA, patient-generated subjective global assessment.

*Indicates a statistically significant difference compared to before intervention, with *p* < 0.05.

### 3.4 Comparison of QoL scores after home-based exercise combined with nutritional care intervention

The home-based exercise combined with nutritional care intervention led to significant improvements in QoL scores in the observation group compared to the control group, as reflected in physical, psychological, social, and material life dimensions. At baseline, there were no statistically significant differences in QoL scores between the observation and control groups across all dimensions (*P* > 0.05). Physical function scores were 73.98 ± 4.12 in the observation group and 75.39 ± 4.24 in the control group (*t* = 1.891, *P* = 0.061). Similar patterns were observed for psychological, social, and material life dimensions, confirming a comparable starting point for both groups. Post-intervention, the observation group exhibited substantial improvements in all QoL dimensions, significantly outperforming the control group (*P* < 0.001 for all indicators). Physical function scores increased to 85.89 ± 5.41 in the observation group compared to 79.00 ± 5.28 in the control group (*t* = 7.233, *P* < 0.001). Psychological function scores rose sharply to 86.49 ± 3.14 in the observation group versus 78.46 ± 3.11 in the control group (*t* = 14.42, *P* < 0.001). Social function scores also improved markedly, reaching 91.12 ± 3.17 in the observation group compared to 83.86 ± 2.41 in the control group (*t* = 14.53, *P* < 0.001). Material life scores showed similar enhancements, increasing to 88.94 ± 3.12 in the observation group compared to 84.49 ± 2.35 in the control group (*t* = 9.079, *P* < 0.001) (Table 4). These findings highlight the effectiveness of the combined intervention in significantly enhancing various aspects of QoL. The integration of resistance-based exercise with tailored nutritional care contributed to notable improvements in physical function, emotional well-being, social interaction, and material satisfaction, emphasizing its clinical relevance in postoperative rehabilitation and recovery.

**TABLE 4:** Comparison of quality of life scores between observation and control groups (mean ± SD).

Indicator	Observation group (*n* = 61)	Control group (*n* = 65)	*t*-value	*p*-value
Physical function (points)	–	–	–	–
Before intervention	73.98 ± 4.12	75.39 ± 4.24	1.891	0.061
After intervention	85.89 ± 5.41*	79.00 ± 5.28*	7.233	<0.001
Psychological function (points)	–	–	–	–
Before intervention	72.65 ± 1.62	73.09 ± 1.55	1.558	0.122
After intervention	86.49 ± 3.14*	78.46 ± 3.11*	14.42	<0.001
Social function (points)	–	–	–	–
Before intervention	78.86 ± 3.21	79.88 ± 3.33	1.748	0.083
After intervention	91.12 ± 3.17*	83.86 ± 2.41*	14.53	<0.001
Material life (points)	–	–	–	–
Before intervention	76.86 ± 2.14	77.51 ± 2.23	1.667	0.098
After intervention	88.94 ± 3.12*	84.49 ± 2.35*	9.079	<0.001

*Indicates a statistically significant difference compared to before intervention, with *p* < 0.05.

### 3.5 Post hoc power analysis

A *post hoc* power analysis was performed using a weighted approach across the five primary outcomes (albumin, prealbumin, transferrin, PG-SGA, and average QoL domain score). The composite effect size (Cohen’s d) was calculated at 2.61, with a corresponding noncentrality parameter of 14.63. The resulting statistical power far exceeded the conventional benchmark of 0.80, demonstrating excellent sensitivity and confirming that the study was adequately powered to detect significant between-group differences with the current sample size.

## 4 Discussion

Sarcopenia, characterized by the progressive loss of muscle mass and strength, poses significant challenges for elderly patients with gastric cancer, a population already burdened by the metabolic and physical demands of malignancy. Malnutrition frequently coexists with sarcopenia in these patients, exacerbating functional decline and compromising recovery. Effective interventions targeting both physical and nutritional deficits are critical to improving clinical outcomes and QoL (13, 14). Traditional hospital-based rehabilitation programs, while effective, often face barriers such as resource limitations and patient adherence. Home-based interventions offer a promising alternative, enabling personalized care in a familiar environment while reducing the burden on healthcare systems. The integration of resistance-based exercises with tailored nutritional care addresses the multifaceted needs of this population. Resistance training stimulates muscle protein synthesis and enhances functional capacity, while personalized nutritional strategies mitigate protein-energy malnutrition and improve metabolic recovery (15–17). The results of this study demonstrate the significant efficacy of a home-based exercise combined with nutritional care intervention in improving nutritional indicators, PG-SGA scores, and QoL among elderly patients with gastric cancer and sarcopenia. The intervention not only addressed nutritional deficiencies but also enhanced physical and psychological function, underscoring its potential as a comprehensive strategy for postoperative rehabilitation.

The observed improvements in albumin, prealbumin, and transferrin levels in the observation group reflect enhanced protein metabolism and better overall nutritional recovery. Albumin, a key marker of protein status, is critical for maintaining oncotic pressure and transporting nutrients. Its significant increase in the observation group compared to the control group suggests that the combined intervention effectively addressed protein-energy malnutrition, a common challenge in patients with sarcopenia and cancer. Prealbumin and transferrin, which are sensitive to short-term nutritional changes, also showed marked improvement, highlighting the role of personalized nutritional support in promoting rapid recovery. The tailored nutritional care component of the intervention likely played a pivotal role in these outcomes. The five-step nutritional therapy model stratified patients according to their needs, ensuring precise and effective supplementation. By addressing specific deficiencies through total parenteral nutrition, enteral nutrition, or oral supplementation, the intervention supported protein synthesis and mitigated catabolic effects associated with cancer and sarcopenia. Additionally, the emphasis on dietary education ensured sustained adherence to nutritional plans, facilitating long-term benefits (18, 19).

The significant reduction in PG-SGA scores in the observation group further underscores the intervention’s efficacy. This improvement likely stems from the synergistic effects of resistance-based exercise and nutritional care. Resistance training is known to counteract muscle wasting by stimulating protein synthesis and increasing muscle mass. The targeted exercises in this study, which included movements such as straight arm chest expansions and leg presses, likely enhanced muscle strength and functional capacity, contributing to better overall nutritional status as reflected in PG-SGA scores. Moreover, improved nutritional intake supported by the intervention addressed energy deficits and replenished critical nutrients, enabling patients to recover more effectively (20, 21). The dual approach of combining exercise and tailored nutrition not only alleviated sarcopenia-related symptoms but also enhanced metabolic efficiency, further contributing to the observed reductions in PG-SGA scores.

The marked improvements in QoL scores across all dimensions—physical, psychological, social, and material life—highlight the holistic impact of the intervention. The enhancement in physical function can be attributed to the resistance-based exercise regimen, which improved mobility, strength, and endurance. These benefits likely translated into greater independence and reduced fatigue, contributing to better physical QoL outcomes. Psychological function improvements may be linked to the intervention’s structured nature, which provided patients with a sense of control and purpose during their recovery. Additionally, regular exercise has well-documented benefits in alleviating anxiety and depression, further enhancing emotional well-being. Social function improvements, as reflected in higher scores, may stem from patients’ increased confidence and physical capability, enabling better engagement in social activities. Material life scores also improved significantly, likely due to the comprehensive educational component of the intervention. By empowering patients with knowledge about nutrition and recovery, the intervention enhanced their ability to make informed decisions, ultimately improving their perceived quality of life in material aspects (22, 23).

These findings underscore the importance of integrating home-based exercise with personalized nutritional care for elderly patients with gastric cancer and sarcopenia. The observed improvements in nutritional indicators, PG-SGA scores, and QoL highlight the intervention’s potential to address the multifaceted challenges faced by this population. This approach offers a scalable, patient-centered strategy that can be implemented in diverse clinical settings to enhance recovery and long-term outcomes. This study has several limitations. First, the sample size, though sufficient for initial analysis, may limit the generalizability of the findings. Additionally, as a retrospective study, physical activity levels outside the intervention were not assessed, limiting the evaluation of their impact on outcomes. The short follow-up period may not fully capture the long-term sustainability of the intervention’s benefits, and variability in patient adherence may have influenced the results. While the study showed improvements in nutritional biomarkers, the underlying physiological mechanisms, particularly how resistance training and nutritional care modulate protein metabolism, inflammation, and catabolic pathways, remain unclear. Future research should investigate these mechanisms to better understand the observed changes. Moreover, the long-term sustainability of the benefits, especially in maintaining muscle mass, requires further investigation, including strategies to promote long-term adherence to home-based interventions. Larger, multicenter studies with extended follow-up are needed to validate these findings and optimize interventions by exploring molecular mechanisms and tailoring strategies to individual patient profiles.

## 5 Conclusion

In conclusion, the home-based exercise combined with nutritional care intervention represents a highly effective strategy for improving nutritional status, functional capacity, and overall quality of life in elderly patients with gastric cancer and sarcopenia, demonstrating its critical role in comprehensive cancer care.

## Data Availability

The raw data supporting the conclusions of this article will be made available by the authors, without undue reservation.
